# ASIC1 and ASIC3 contribute to acidity-induced EMT of pancreatic cancer through activating Ca^2+^/RhoA pathway

**DOI:** 10.1038/cddis.2017.189

**Published:** 2017-05-18

**Authors:** Shuai Zhu, Hai-Yun Zhou, Shi-Chang Deng, Shi-Jiang Deng, Chi He, Xiang Li, Jing-Yuan Chen, Yan Jin, Zhuang-Li Hu, Fang Wang, Chun-You Wang, Gang Zhao

**Affiliations:** 1 Department of Pancreatic Surgery, Pancreatic Disease Institute, Union Hospital, Tongji Medical College, Huazhong University of Science and Technology, Wuhan, China; 2 Department of Pharmacology, School of Basic Medicine, Tongji Medical College, Huazhong University of Science and Technology, Wuhan, China; 3 Department of Gastrointestinal Surgery, Union Hospital West Campus, Tongji Medical College, Huazhong University of Science and Technology, Wuhan, China

## Abstract

Extracellular acid can have important effects on cancer cells. Acid-sensing ion channels (ASICs), which emerged as key receptors for extracellular acidic pH, are differently expressed during various diseases and have been implicated in underlying pathogenesis. This study reports that ASIC1 and ASIC3 are mainly expressed on membrane of pancreatic cancer cells and upregulated in pancreatic cancer tissues. ASIC1 and ASIC3 are responsible for an acidity-induced inward current, which is required for elevation of intracellular Ca^2+^ concentration ([Ca^2+^]i). Inhibition of ASIC1 and ASIC3 with siRNA or pharmacological inhibitor significantly decreased [Ca^2+^]i and its downstream RhoA during acidity and, thus, suppressed acidity-induced epithelial–mesenchymal transition (EMT) of pancreatic cancer cells. Meanwhile, downregulating [Ca^2+^]i with calcium chelating agent BAPTA-AM or knockdown of RhoA with siRNA also significantly repressed acidity-induced EMT of pancreatic cancer cells. Significantly, although without obvious effect on proliferation, knockdown of ASIC1 and ASIC3 in pancreatic cancer cells significantly suppresses liver and lung metastasis in xenograft model. In addition, ASIC1 and ASIC3 are positively correlated with expression of mesenchymal marker vimentin, but inversely correlated with epithelial marker E-cadherin in pancreatic cancer cells. In conclusion, this study indicates that ASICs are master regulator of acidity-induced EMT. In addition, the data demonstrate a functional link between ASICs and [Ca^2+^]i/RhoA pathway, which contributes to the acidity-induced EMT.

Pancreatic cancer is one of the most aggressive cancers with a 5-year survival rate of about 5% and a median survival rate of about 6 months.^1^ This poor prognosis and ultimate cause of death are mainly due to metastatic invasion. Strikingly, recent studies suggest that pancreatic cancer metastasizes very early in their development.^2^ Therefore, it is critical to identify the underlying molecular mechanisms of invasion and metastasis of pancreatic cancer.^3^ Epithelial–mesenchymal transition (EMT) has a major role in tumor invasion and metastasis. In the process of EMT, the cells lose epithelial characteristics and acquire invasive properties and stem cell-like features.^4^ EMT is associated with poor survival in surgically resected pancreatic cancer.^5^ Signaling molecules, such as Snail, which have an essential role during EMT, promote metastatic process of pancreatic cancer.^6^ Specifically, our previous study reveals that both chronic pancreatitis and pancreatic cancer demonstrated active EMT process, which also indicates the pivotal role of EMT in tumorigenesis of pancreatic cancer.^7^ However, the precise mechanism for initiation of EMT in pancreatic cancer has been not elucidated fully.

Recent studies have noted that the acidity is an intrinsic hallmark of tumor microenvironment. Owing to the ‘Warburg effect’, which is an intrinsic characteristic in malignant cells, metabolism shifts to glycolysis even when there is enough O_2_. This shift remarkably increases glucose metabolism, which leads to a rapid production of lactic acids.^8^ Moreover, the insufficient perfusion and chaotic vascular structure of tumor further increase the accumulation of lactic acid and H^+^.^9^ Thus, malignant tumors display an acidic extracellular pH (pH_e_) when compared with normal tissue under physiological conditions.^10, 11, 12^ Interestingly, extracellular acidity as a favoring factor of tumor progression and metastatic dissemination is involved in EMT. Melanoma cells, which are exposed to acidic extracellular environment, display a significant EMT profile and an increased invasiveness *in vitro* and *in vivo*.^13^ Acidic extracellular pH induces the expression of EMT regulator, including ZEB1, Twist1 and Twist2, accompanied with enhancing cell motility and invasion in lung carcinoma.^14^ Meaningfully, our previous work reveals that acidic microenvironment induces EMT of pancreatic cancer cells through regulating miR-652/ZEB1 pathway.^15^ In this study, we work to investigate how the acidic extracellular pH, a chemical signaling of metabolic microenvironment, is detected by pancreatic cancer cells and further promotes the transduction of EMT as biological signaling.

Acid-sensing ion channels (ASICs), an H^+^-gated subgroup of the epithelial Na^+^ channel/degenerin (ENaC/DEG) superfamily, are triggered by increased extracellular acidification.^16, 17^ To date, six ASIC subunits have been identified: ASIC1a, ASIC1b, ASIC2a, ASIC2b, ASIC3 and ASIC4.^18^ The pH values required for half-maximal activation of ASICs (pH_0.5_) are varied from 4.9 to 6.8.^19^ ASICs are involved in most of the acidity-associated physiological and pathological functions in nervous system disease.^20, 21^ It has been reported that high-grade glioma cells express voltage-independent, amiloride-inhibitable inward Na^+^ current, which is attributed to mixed ASIC1 and ASIC2, but not exists in normal astrocyte or low-grade glioma. Moreover, inhibition of this conductance decreases the growth and migration of glioma cells.^22^ Other research has shown that ASIC1 is highly expressed in D54-MG human glioblastoma multiform cells than that of primary human astrocytes. Knockdown of ASIC1 or using pharmacological inhibitor effectively abolishes the whole-cell patch-clamp current and inhibits tumor migration in glioma cells.^23^ Given that ASIC1 and ASIC3 are the most susceptible to acidic pH_e_ and function as acidic pH sensors,^19^ we investigate whether ASIC1 and ASIC3 are expressed in pancreatic cancer and contribute to acidity-induced EMT of pancreatic cancer.

## Results

### ASIC1 and ASIC3 are functionally expressed on pancreatic cancer cells

**Figure 1:**
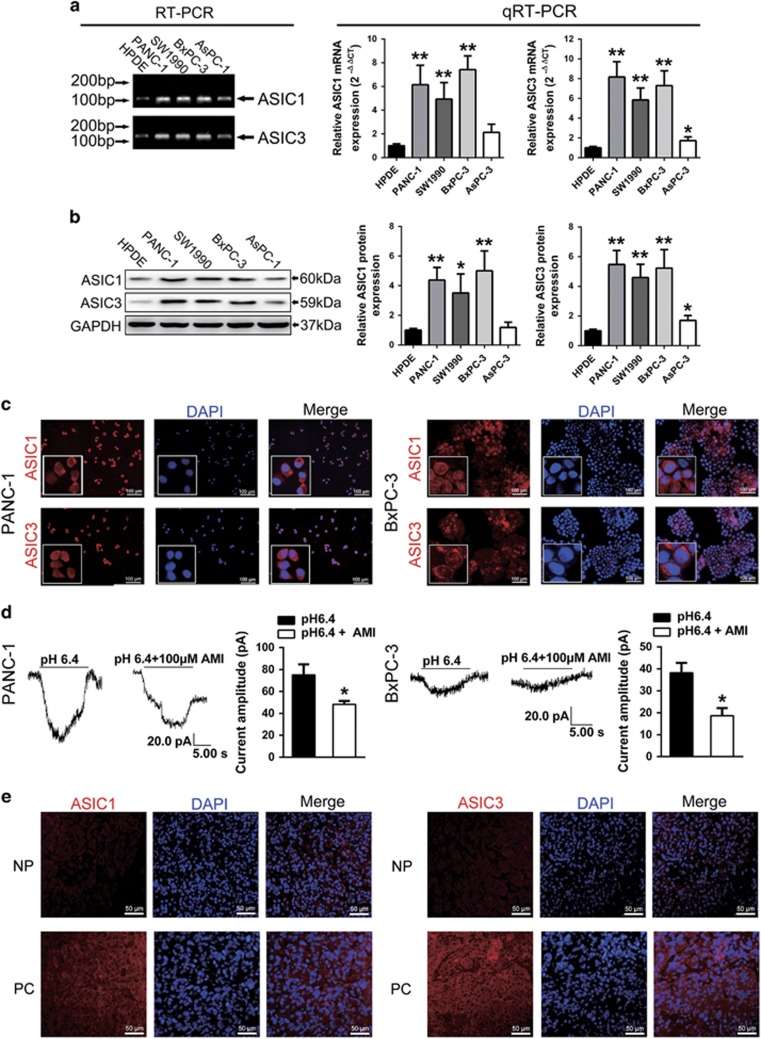
ASIC1 and ASIC3 are functionally expressed in pancreatic cancer. (**a** and **b**) The mRNA and protein expression of ASIC1 and ASIC3 in four pancreatic cancer cell lines (PANC-1, SW1990, BxPC-3 and AsPC-1) and normal pancreatic ductal cells (HPDE) were measured by RT-PCR, qRT-PCR and western blot. Values were normalized against HPDE from three experiments performed in triplicate and were presented as means±S.D. (*P<0.05; **P<0.01). (**c**) Representative immunofluorescence of ASIC1 and ASIC3 in PANC-1 and BxPC-3 cells. (**d**) Whole-cell current of PANC-1 and BxPC-3 incubated with acidic (pH 6.4) extracellular solution was recorded by whole-cell voltage-clamp recording. After cells were treated with amiloride, whole-cell current was recorded again. (**e**) Representative immunofluorescence of ASIC1 and ASIC3 in pancreatic cancer (PC) tissues and its paired adjacent noncancerous pancreatic (NP) tissues from patients

It has been well demonstrated that ASICs are involved in numerous physiological and pathological process in nervous system, whereas there is rare research about the expression and function of ASICs in non-nervous system. Given that ASIC1 and ASIC3 are the most sensitive to acidic pH_e_ and function as acidic pH sensors, we first detected the expression of ASIC1 and ASIC3 in pancreatic cancer cells. Reverse transcription-PCR (RT-PCR) and quantitative real-time RT-PCR (qRT-PCR) displayed that the mRNA of ASIC1 and ASIC3 in pancreatic cancer cell lines (PANC-1, SW1990, BxPC-3 and AsPC-1) significantly increased compared with normal pancreatic ductal cells (HPDE) (Figure 1a). Western blot confirmed that the protein of ASIC1 and ASIC3 in pancreatic cancer cell lines was increased compared with normal pancreatic ductal cells (Figure 1b). Immunofluorescence assay showed that the fluorescence was mainly expressed in the membrane, validating that proteins of ASIC1 and ASIC3 are expressed in PANC-1 and BxPC-3 cell lines (Figure 1c).

Based on the above results, we investigated physiological properties of ASICs in HPDE, PANC-1 and BxPC-3 cells. Acidic solution did not induce inward currents in HPDE (Supplementary Figure S1A). However, PANC-1 and BxPC-3 cells appeared an inward current immediately when the pH of perfusion solution was switched from 7.4 to 6.4 (Figure 1d). The current amplitude is 75.09±9.623 pA on PANC-1 and 38.20±4.501 pA on BxPC-3. Interestingly, compared with classical ASICs currents, the currents detected on both cell lines were not so rapid rising and with longer decay time. However, these inward currents were inhibited with ASIC inhibitor amiloride (100 *μ*M). After treated with amiloride, the current amplitude reduced to 48.17±3.178 pA on PANC-1 and 18.66±1.994 pA on BxPC-3.

In consistence with the results in pancreatic cancer cell lines, the immunofluorescence assay showed that ASIC1 and ASIC3 in pancreatic cancer specimens were increased compared with paired adjacent noncancerous tissues (Figure 1e). Therefore, these results indicate that ASIC1 and ASIC3 are functionally expressed in pancreatic cancer cells.

### Inhibition of ASIC1 and ASIC3 suppresses acidity-promoted invasion and migration of pancreatic cancer cells

**Figure 2:**
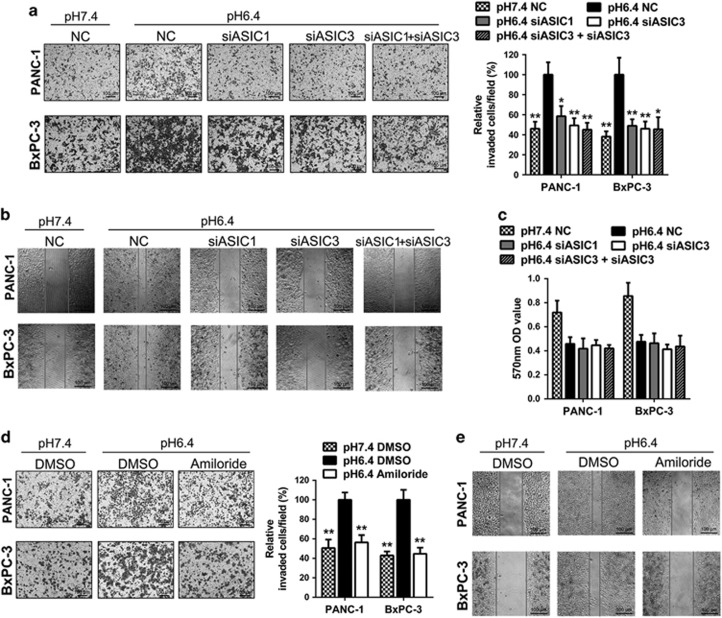
Inhibition of ASIC1 and ASIC3 suppresses acidity-promoted invasion and migration of pancreatic cancer cells. PANC-1 and BxPC-3 were transfected with negative control (NC) or siRNA of ASIC1 and ASIC3 (siASIC1 and siASIC3) and cultured in pH 7.4 or pH 6.4 medium as indicated for 48 h. (**a**) The invasive ability was evaluated by Transwell assay. The histogram showed the percentage of invasion cells per field when compared with negative control cultured in pH 6.4 medium. (**b**) The migration ability was measured by wound-healing assay. (**c**) The cell viability was measured by MTT assay. PANC-1 and BxPC-3 were treated with amiloride and DMSO, and cultured in pH 7.4 or pH 6.4 medium as indicated for 48 h. (**d**) The invasive ability was evaluated by Transwell assay. The histogram showed the percentage of invasion cells per field when compared with negative control cultured in pH 6.4 medium. (**e**) The migration ability was measured by wound-healing assay. Experiments were performed three times in triplicate and were presented as means±S.D. (*P<0.05; **P<0.01)

In order to evaluate the role of ASIC1 and ASIC3 in acidity-promoted invasion and migration, the expression of ASIC1 and ASIC3 was downregulated with siRNA. The si-2 of ASIC1 siRNA and si-2 of ASIC3 siRNA had the most distinct restraining effect on the expression of mRNA and protein, respectively (Supplementary Figures S2A and B). After knockdown of ASIC1 and ASIC3 separately or simultaneously (Supplementary Figure S2C), the acidity-promoted invasion and migration of pancreatic cancer cells were inhibited (Figures 2a and b). However, simultaneous inhibition of ASIC1 and ASIC3 did not have synergistic effect. Meanwhile, knockdown of ASIC1 and ASIC3 with siRNA did not display remarkable effect on viability of PANC-1 and BxPC-3 cells (Figure 2c). Thus, the results of Transwell assay and wound-healing test were attributed to the decreased activity of invasion and migration but not proliferation inhibition. Furthermore, amiloride, a nonspecific inhibitor to ASICs, distinctly attenuated the acidity-promoted invasion and migration of PANC-1 and BxPC-3 cells (Figures 2d and e). Similarly, the specific inhibitor for ASIC1, PcTx1 demonstrated inhibitory effects to acidity-promoted invasion and migration of pancreatic cancer cells (Supplementary Figures S3A and B). These results suggest that ASIC1 and ASIC3 have an important role in acidity-promoted invasion and migration.

### Inhibition of ASIC1 and ASIC3 suppresses acidity-promoted EMT of pancreatic cancer

**Figure 3:**
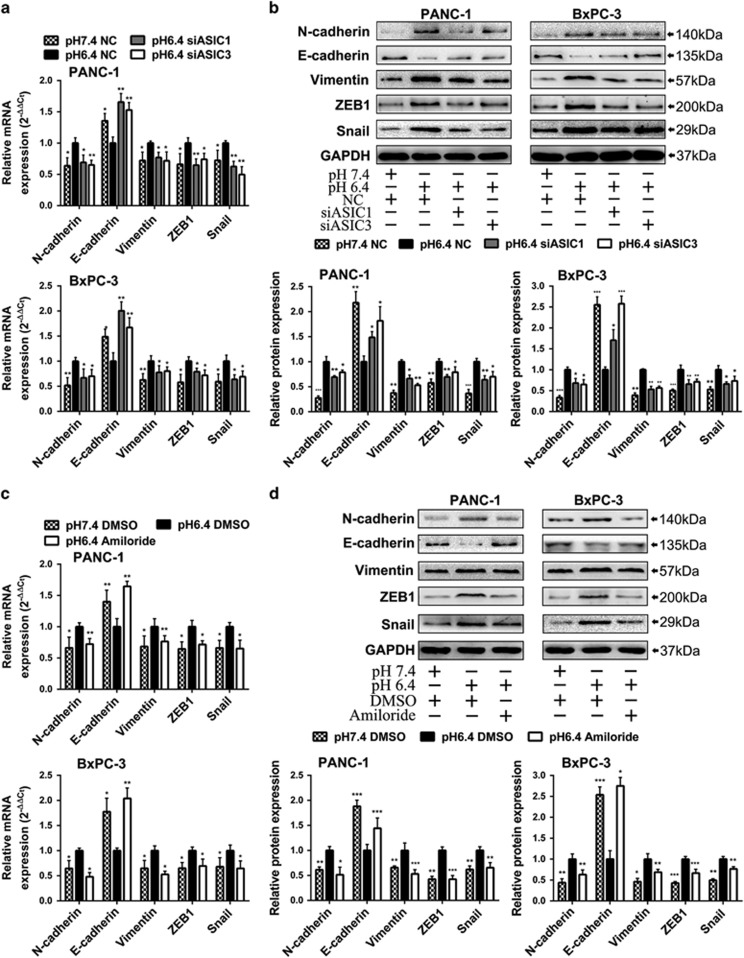
Knockdown of ASIC1 and ASIC3 suppresses acidity-induced EMT of pancreatic cancer cells. (**a** and **b**) PANC-1 and BxPC-3 were transfected with negative control (NC) or siRNA of ASIC1 and ASIC3 (siASIC1 and siASIC3) and cultured in pH 7.4 or pH 6.4 medium as indicated for 48 h. The mRNA and protein expression of N-cadherin, E-cadherin, Vimentin, ZEB1, Snail were measured by qRT-PCR and western blot. (**c** and **d**) PANC-1 and BxPC-3 were treated with amiloride and DMSO, and cultured in pH 7.4 or pH 6.4 medium as indicated for 48 h. The mRNA and protein expression of N-cadherin, E-cadherin, Vimentin, ZEB1, Snail were measured by qRT-PCR and western blot. Values were normalized against negative control in pH 6.4 medium. Experiments were performed three times in triplicate and are presented as means±S.D. (*P<0.05; **P<0.01; ***P<0.001)

As our previous results showed that the enhanced migration and invasion of pancreatic cancer cell in acidity were attributed to the acidity-induced EMT, we further investigated whether ASIC1 and ASIC3 contributed to the acidity-induced EMT. Our results showed that knockdown of ASIC1 or ASIC3 impaired acidity-induced mesenchymal profile change in PANC-1 and BxPC-3 cells (Supplementary Figure S4A), accompanied with the decreased expression of the mesenchymal marker, such as Vimentin, N-cadherin, Snail and ZEB1. On the contrary, the expression of epithelial marker E-cadherin was obviously increased in both mRNA and protein level (Figures 3a and b). Similarly, amiloride also restrained the acidity-induced EMT, presenting with mesenchymal to epithelial transition (MET) (Supplementary Figure S4B), downregulation of Vimentin, N-cadherin, Snail and ZEB1, as well as upregulation of E-cadherin in PANC-1 and BxPC-3 cells (Figures 3c and d). Coincidently, PxTC1 reverted the acidity-induced alteration of Vimentin, N-cadherin, Snail, ZEB1 and E-cadherin (Supplementary Figures S5A and B). Thus, these results indicate that ASIC1 and ASIC3 are involved in the acidity-induced EMT of pancreatic cancer cells.

### Overexpression of ASIC1 or ASIC3 promoted EMT of AsPC-1 in acidic condition

We have showed that the expression of ASIC1 and ASIC3 in AsPC-1 were relatively lower than that of PANC-1 and BxPC-3, therefore, we upregulated the expression of ASIC1 and ASIC3 in AsPC-1 (Supplementary Figure S6A) to further determine their role in EMT. As expected, overexpression of ASIC1 and ASIC3 promoted the invasion and migration of AsPC-1 under acidic condition (Supplementary Figures S6B and C). Correspondingly, overexpression of ASIC1 and ASIC3 increased the expression of Vimentin, N-cadherin, Snail and ZEB1, decreased the expression of E-cadherin during acidity (Supplementary Figures S6D and E).

### The elevation of [Ca^2+^]i is involved in acidity-induced EMT of pancreatic cancer cells

**Figure 4:**
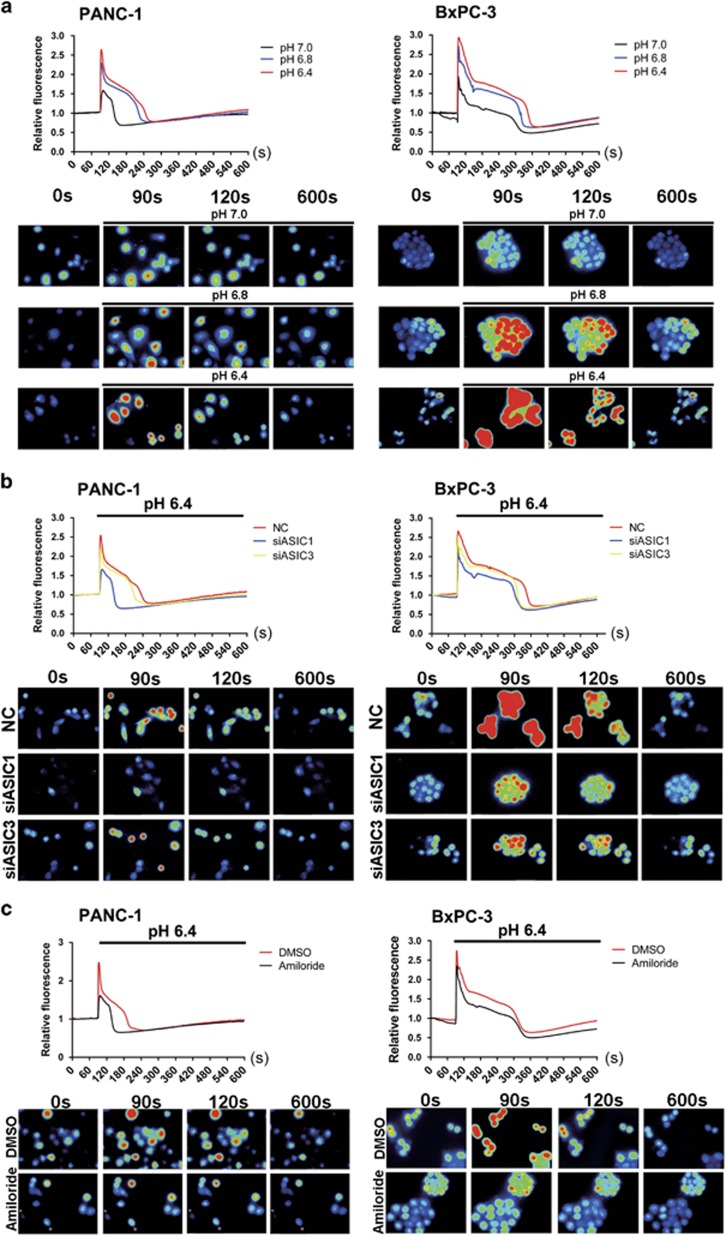
The acidity-induced elevation of [Ca^2+^]i is suppressed by inhibition of ASIC1 and ASIC3. (**a**) Calcium imaging detected dynamic change in [Ca^2+^]i when PANC-1 and BxPC-3 cells were treated with different acidic pH medium at 90 s. Pictures showed [Ca^2+^]i indicated by Fura-2/AM at indicated time. (**b** and **c**) After inhibition of ASIC1 and ASIC3 by siRNA or amiloride, calcium imaging detected dynamic change in [Ca^2+^]i of PANC-1 and BxPC-3 cells in acidic medium (pH 6.4). Pictures showed intracellular Ca^2+^ concentration indicated by Fura-2/AM at indicated time. Experiments were performed three times

It has been demonstrated that the Ca^2+^ permeability of ASICs provides a voltage-independent pathway for Ca^2+^ entry into cells.^16^ Moreover, the intracellular Ca^2+^ concentration ([Ca^2+^]i) is ubiquitous second messenger and acts as a crucial regulator of cell migration, which has a profound role in tumor metastasis.^24^ We therefore wondered whether ASIC1 and ASIC3 may contribute to the acidity-induced EMT through regulating [Ca^2+^]i. Compared with normal pH, the calcium imaging assay displayed obvious elevation of [Ca^2+^]i of PANC-1 and BxPC-3 cells in pH-dependent manner (Figure 4a). Meantime, this acid-promoted elevation of [Ca^2+^]i was significantly attenuated by knockdown of ASIC1 or ASIC3 (Figure 4b), as well as blocked by pretreatment with amiloride (Figure 4c). These results suggest that acidic microenvironment evokes elevation of [Ca^2+^]i though activation of ASIC1 and ASIC3.

It has been demonstrated that the elevated [Ca^2+^]i facilitates the acidity-promoted EMT. We therefore used calcium chelating agent BAPTA-AM to decrease [Ca^2+^]i concentration in pancreatic cancer cells. Pretreatment with BAPTA-AM significantly restrained invasion and migration of PANC-1 and BxPC-3 cells in acidity condition (Supplementary Figures S7A and B). Compared with negative control, BAPTA-AM eliminated the morphologic transformation induced by acidity in PANC-1 and BxPC-3 cells (Supplementary Figure S7C), which was evidenced by decreased mesenchymal marker and increased epithelial marker in acidity conditions (Supplementary Figures S7D and E). Together, these findings provide compelling evidence that ASIC1 and ASIC3-[Ca^2+^]i signaling pathway confers EMT capacity to pancreatic cancer cells in acidity condition.

### ASIC1/3-[Ca^2+^]i signaling pathway facilitates acidity-induced EMT through upregulation of RhoA activity

**Figure 5:**
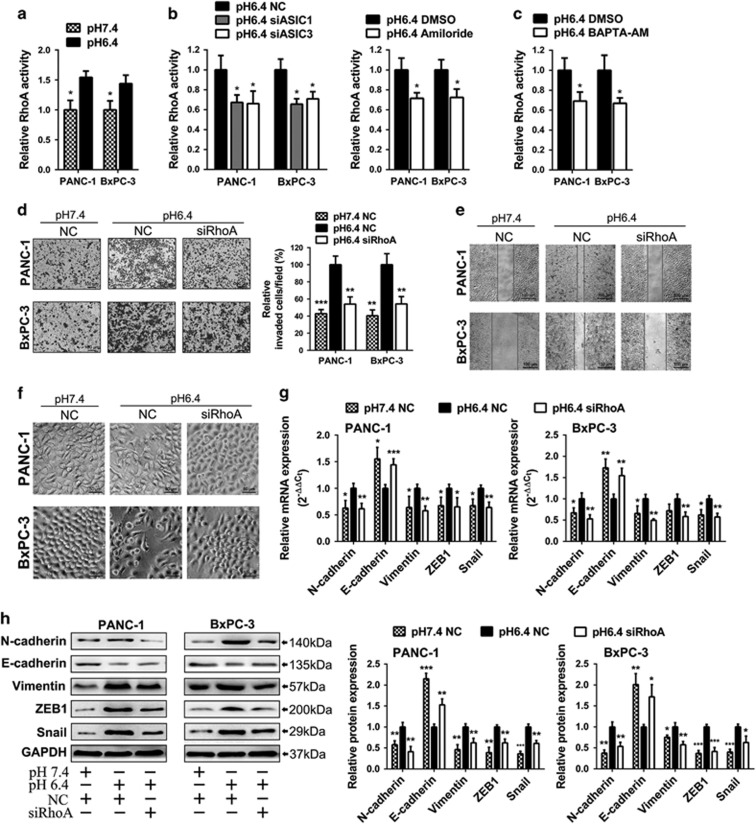
ASIC1/ASIC3-[Ca^2+^]i signaling pathway upregulates RhoA activity of pancreatic cancer cells in acidity condition. (**a**) RhoA activity (GTP-bound RhoA) was detected in PANC-1 and BxPC-3 treated in pH 6.4 or pH 7.4 medium as indicated for 48 h. (**b**) After inhibition of ASIC1 and ASIC3 by siRNA or amiloride, the RhoA activity was detected in PANC-1 and BxPC-3 cells, which were incubated acidity medium (pH 6.4) for 48 h. (**c**) With pretreatment of BAPTA-AM or DMSO for 1 h, RhoA activity was detected in PANC-1 and BxPC-3 cells after cells were incubated with acidic medium (pH 6.4) for 48 h. PANC-1 and BxPC-3 were transfected with negative control (NC) or siRNA of RhoA (siRhoA) and cultured in pH 7.4 or pH 6.4 medium as indicated for 48 h. (**d**) The invasive ability was evaluated by Transwell assay and the histogram showed the percentage of invasion cells per field when compared with negative control cultured in pH 6.4 medium. (**e**) The migration ability was measured by wound-healing assay. (**f**) The representative pictures of cellular morphology. (**g**) The mRNA of N-cadherin, E-cadherin, Vimentin, ZEB1, Snail was measured by qRT-PCR. (**h**) The protein of N-cadherin, E-cadherin, Vimentin, ZEB1, Snail was measured by western blot. Values were normalized against negative control in pH 6.4 medium. Experiments were performed three times in triplicate and are presented as means±S.D. (*P<0.05; **P<0.01; ***P<0.001)

We next determined how [Ca^2+^]i contributes to acidity-induced EMT. As [Ca^2+^]i has been reported to activate RhoA that has a crucial role in actin cytoskeletal rearrangement and cell migration,^25, 26^ we first compared the activity of RhoA in acidity with normal condition. As shown in Figure 5a, the activity of RhoA was obviously elevated in PANC-1 and BxPC-3 cells treated with acidity. In contrast, inhibition of ASIC1 and ASIC3 by siRNA or amiloride distinctly decreased RhoA activity in acidity (Figure 5b). Also, Chelating [Ca^2+^]i with BAPTA-AM reduced RhoA activity that was induced by acidity (Figure 5c).

We then assessed whether RhoA was important for ASIC1/3-[Ca^2+^]i signaling pathway-induced EMT in acidity condition. RhoA expression was knockdown by RNAi, and the si-3 showed the most obvious inhibitory effect on mRNA and protein expression of RhoA among all three siRNA (Supplementary Figure S8A). We found that RhoA knockdown significantly repressed acidity-induced migration and invasion of pancreatic cancer cells (Figures 5d and e), supporting a role of RhoA in acidity-induced EMT. The transition of mesenchymal morphology induced by acidity was rescued by RhoA siRNA (Figure 5f). Furthermore, knockdown of RhoA reversed the expression of EMT marker (Figures 5g and h). Together, these results indicate that RhoA is involved in the EMT of pancreatic cancer cells induced by ASIC1/3-[Ca^2+^]i activation in acidic microenvironment, highlighting that RhoA is a major effector of acidity-induced EMT of pancreatic cancer.

### Knockdown of ASIC1 and ASIC3 inhibits metastasis of pancreatic cancer cells *in vivo*

**Figure 6:**
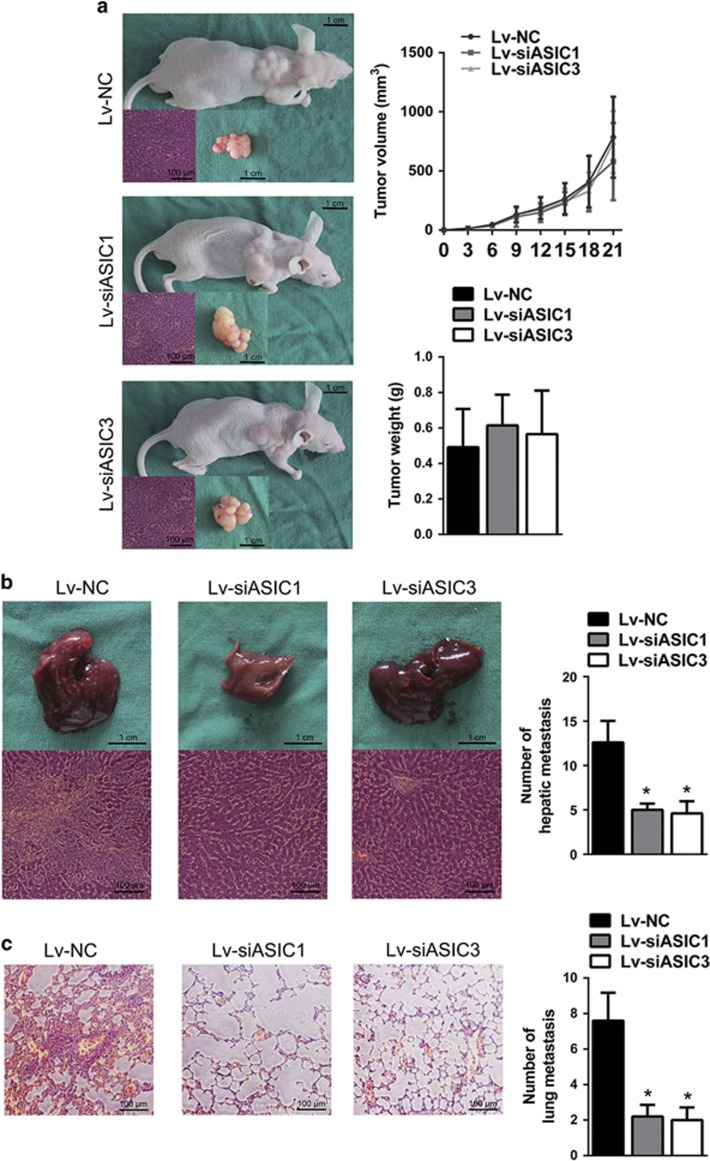
Knockdown of ASIC1 and ASIC3 inhibits metastasis of pancreatic cancer cells *in vivo*. (**a**) Photographs of nude mice and tumor showed subcutaneous xenograft of BxPC-3, which was transfected with lentivirus containing sequence of ASIC1 siRNA, ASIC3 siRNA or negative control (Lv-siASIC1, Lv-siASIC3 and Lv-NC). H&E stain confirmed tumor formation. Graph represented tumor volumes at the indicated days during the experiment and tumor weight after mice were killed. (**b**) Pictures of liver and H&E stain showed liver metastases of subcutaneous xenograft. The histogram showed average number of live metastases in each group. (**c**) H&E stain showed lung metastases of subcutaneous xenograft. The histogram showed average number of lung metastases in each mouse. The data represent the mean±S.D. (*P<0.05)

To further demonstrate that ASIC1/3 facilitated aggressive behavior of pancreatic cancer cells *in vivo*, we next used BxPC-3 cells to establish tumor xenografts with stable knockdown of ASIC1 and ASIC3, respectively. Supporting the above results *in vitro*, knockdown of ASIC1 and ASIC3 in acidity-pretreated cells had no obvious effect on the growth of implanted tumor (Figure 6a). Nevertheless, the liver and lung metastasis was significantly decreased after downregulation of ASIC1 and ASIC3, respectively, which was confirmed by the H&E-stained slides (Figures 6b and c). Taken together, our results provide strong evidence that ASIC1 and ASIC3 promote metastasis of human pancreatic cancer cells *in vivo*.

### The expression of ASIC1 and ASIC3 are elevated in pancreatic cancer and correlated with the level of EMT marker

**Figure 7:**
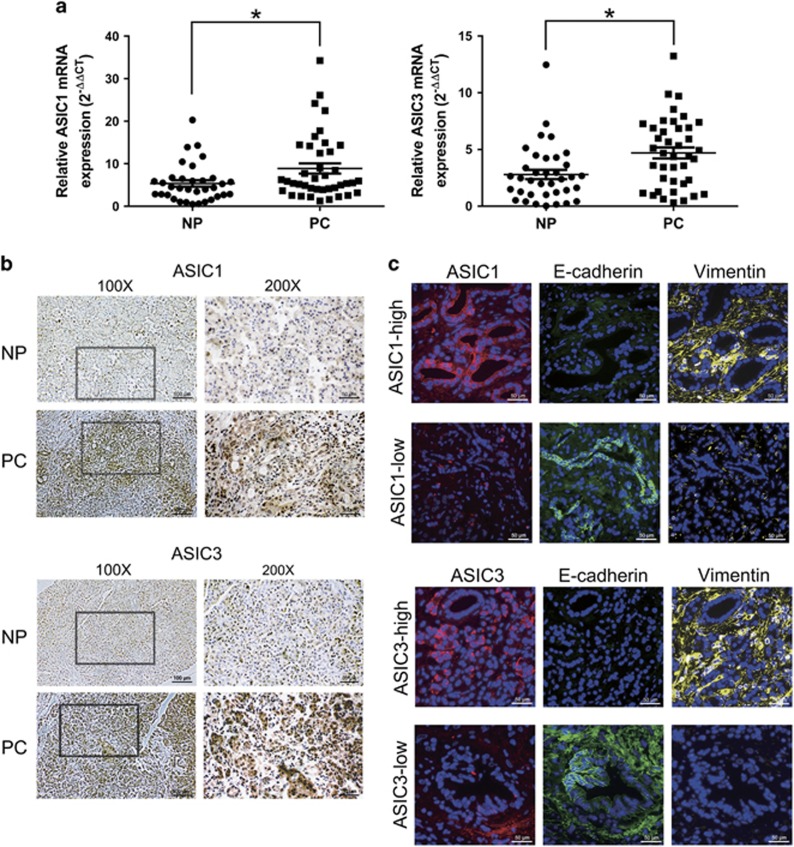
ASIC1 and ASIC3 are overexpressed and correlated to EMT marker in pancreatic cancer. (**a**) The mRNA expression of ASIC1 and ASIC3 in pancreatic cancer (PC) tissues when compared with matched adjacent noncancerous pancreatic (NP) tissues. Values were normalized against negative control in pH 6.4. Experiments were performed three times in triplicate and are presented as means±S.D. (*P<0.05). (**b**) Representative picture of immunohistochemical analysis showed the expression of ASIC1 and ASIC3 in pancreatic cancer tissues and matched adjacent noncancerous pancreatic tissues. (**c**) The representative immunofluorescence of ASIC1 (red), ASIC3 (red), E-cadherin (green), vimentin (yellow) and DAPI (blue) in pancreatic cancer tissues

To further confirm the effects of ASIC1/3 on EMT, we next assessed the expression of ASIC1/3 and EMT marker in pancreatic cancer tissues. As shown in Figure 7a, ASIC1 and ASIC3 mRNA in pancreatic cancer tissues were significantly higher than that in paired noncancerous pancreatic tissues. Moreover, ASIC1 and ASIC3 were expressed in all 40 pancreatic cancer tissues, but undetected in 6 and 4 out of 40 pancreatic noncancerous tissues, respectively. The higher expression of ASIC1/ASIC3 was associated with poor tumor differentiation, advanced TNM stage, lymph node metastasis and distant metastasis (Supplementary Table S2 and S3). Immunohistochemical results showed that ASIC1 or ASIC3 were much more extensively expressed in pancreatic cancer tissues than that in paired adjacent noncancerous tissues (Figure 7b). Furthermore, the immunofluorescence assay demonstrated that ASIC1 and ASIC3 positively correlated with the expression of mesenchymal marker Vimentin, inversely correlated with epithelial marker E-cadherin in human pancreatic cancer samples (Figure 7c).

## Discussion

Acidic microenvironment is an inherent characteristics of tumor, which facilitates aggressive invasion and metastasis in carcinogenesis.^27^ Previous research has shown that acidic condition stimulates tumor cell invasion and this may involve lysosomal proteases, promotion of normal cell death and extracellular matrix degradation.^28^ However, the precise mechanism by which extracellular acidic pH signal regulates invasion and metastasis of tumor cells has not been well defined. The most important new finding in this study is that ASIC1 and ASIC3 transduce the acidic pH to elevation of [Ca^2+^]i concentration and RhoA activity, promoting EMT of pancreatic cancer cells during acidic microenvironment. Our study elaborates an integrated signaling pathway to account for the effect of acidic microenvironment on invasion and metastasis of pancreatic cancer.

Ion channels are emerging as a crucial link in the cross-talk between tumor cells and their microenvironment. Ion channels are in a strategic position to sense and transmit extracellular signals into the intracellular machinery,^29, 30^ which serve as a basis for each major step of the metastatic cascade including EMT.^31, 32^ So far, the role of ASIC1 and ASIC3 in cell migration and proliferation has been mainly shown in astrocytes, glioblastoma and breast cancer, whereas its role in other cancers has not been well elucidated.^22, 23, 33^ Much of our present work stems from ASIC1 and ASIC3 are the most sensitive to acidity in ASICs. This study indicates that ASIC1 and ASIC3 senses the variation of acidic microenvironment and transmits this signal into pancreatic cancer cells, resulting in EMT to enhance migration and invasion. Similarly, research has demonstrated that ASIC1 is overexpressed in hepatocellular carcinoma (HCC) and associated with advanced clinical stage, and knockdown of ASIC1 significantly inhibits cell migration and invasion of HCC.^34^ ASIC1 had a critical role in acidosis-induced reactive oxidative species and NF-*κ*B activation though calcium influx in breast cancer, and ASIC1 inhibitors cause a significant reduction of tumor growth and tumor load.^33^ Differently, other research has shown that ASIC1 is downregulated in clear cell renal cell carcinoma and correlated with higher tumor grades.^35^ Therefore, these results imply that the expression and function of ASICs in various cancers is heterogeneous, which requires further investigation.

[Ca^2+^]i maintains at a low level of approximately 100 nM in resting cells, whereas it can rise globally to in excess of 1 *μ*M in response to stimulation.^36^ Although alterations in Ca^2+^ signaling may not be necessary for the initiation of cancer, the consequences of altered calcium transport in cancer cells are significant and contribute to tumor progression.^37^ For example, transient receptor potential vanilloid type-2 (TRPV2) affects cancer cell aggressiveness by influencing basal [Ca^2+^]i, whereas suppression of TRPV2 inhibits the migration of prostate cancer cells by reducing [Ca^2+^]i.^38^ Moreover, recent works set up an important role for the intracellular calcium signal in the induction of EMT in human cancer cells. Transient receptor potential melastatin-like 7 induces EMT of breast cancer through a transient increased [Ca^2+^]i, and attenuation of this channel reduces epidermal growth factor (EGF) and hypoxia-induced EMT.^39^ Given that ASIC1 and ASIC3 can evoke Ca^2+^ current and elevate [Ca^2+^]i level of particular cells in acidification,^33, 40, 41, 42^ we hypothesize that [Ca^2+^]i acts as the second messenger, which mediates the acidity-induced EMT of pancreatic cancer. This study displayed that acidic pH_e_ significantly increased [Ca^2+^]i of pancreatic cancer cells in a pH-dependent manner, which was repressed by the inhibition of ASIC1 and ASIC3. More importantly, the downregulation of [Ca^2+^]i level with a cell-permeant calcium chelator BAPTA-AM distinctly suppressed acidity-induced EMT of pancreatic cancer cells. Therefore, these findings intensively highlights that the [Ca^2+^]i functioned as the second message to bridge the ASIC1/ ASIC3 and acidity-induced EMT of pancreatic cancer cells.

It has been documented that intracellular calcium homeostasis is involved in the activation of RhoA, which is one of the Rho family of small GTPase. RhoA has a crucial role in controlling cell shape, polarity, invasion and migration.^43^ Calcium channel-induced Ca^2+^ release has a significant role in toning vascular smooth muscle contractility through activation of RhoA/ROCK.^26^ Polyamine-dependent cell migration is partially initiated by the formation of myosin II stress fibers as a result of Ca^2+^-activated RhoA.^44^ Similarly, the elevation mTORC1 and mTORC2 activity promotes EMT, motility and metastasis of colorectal cancer cells via activating RhoA and Rac1 signaling.^25^ On the other hand, F-box and WD repeat domain-containing 7 (Fbxw7) induces apoptosis and growth arrest and inhibits the EMT in partially through downregulation of RhoA signaling.^45^ In our present work, acidic pH_e_ obviously increased RhoA activity of pancreatic cancer cells, which was suppressed by the inhibition of ASIC1 and ASIC3, and chelation of [Ca^2+^]i with BAPTA-AM. Furthermore, knockdown of RhoA with RNAi significantly inhibited the acidity-induced EMT and weakened the invasion and migration of pancreatic cancer cells. Therefore, our results suggest that the ASIC1/3-[Ca^2+^]i signaling pathway, which regulating acidity-induced EMT may be attributed to, at least partially, the activation of RhoA signaling. On the contrary, other study has shown that depletion of RhoA with RNAi leads to the elongated and protrusive phenotype in PC3 prostate cancer cells and MDA-MB-231 breast cancer cells, and therefore correlates with an increase in invasive behavior.^46^ Nevertheless, these variable consequences of RhoA imply that the role of RhoA for cell migration, invasion and EMT is heterogeneous and has a strong dependence on cell background.^47^

Collectively, our study highlights a mechanism for acidity-induced EMT in pancreatic cancer cells through ASIC1/3-[Ca^2+^]i-RhoA axis, which contributed to the metastasis of pancreatic cancer. Therefore, ASIC1 and ASIC3 may represent exciting targets for therapeutic intervention in pancreatic cancer. Alternatively, targeting [Ca^2+^]i or its downstream effector RhoA may offer more tractable treatment strategy.

## Materials and Methods

### Patients and tumor tissues

A total of 40 self-pair of tumors and matched noncancerous tissues were derived from patients undergoing pancreatectomy or biopsy before chemotherapy at Pancreatic Disease Institute of Union Hospital (Wuhan, China). Those patients were performed with pancreatectomy or palliative surgery including I^125^ seeds implantation, as well as choledochojejunostomy and gastroenterostomy depending on the National Comprehensive Cancer Network (NCCN) guideline for pancreatic cancer. Pathologic analysis was performed to confirm the diagnosis of patients and histopathology of the specimens. Samples used for mRNA detection were immediately snap-frozen and stored at −80 °C until extraction of RNA. Sample used for immunohistochemistry and immunofluorescence were fixed by formalin and embedded by paraffin. Informed consent was acquired from all patients and the study was approved by the ethics committee of Huazhong University of Science and Technology, Wuhan, China.

### Cell culture

The human pancreatic cancer cell lines (PANC-1, BxPC-3, SW1990 and AsPC-1) and normal pancreatic ductal cells (HPDE) were purchased from American Type Culture Collection (South San Francisco, USA) in 2014 and they were tested and authenticated for genotypes by DNA fingerprinting. These cell lines were passaged for <6 months after resuscitation. Cell lines were cultured in RPMI-1640 medium (HyClone, Logan, UT, USA) supplemented with 10% fetal bovine serum (HyClone), 100 U/ml penicillin and 100 *μ*g/ml streptomycin under a humidified 5% CO_2_, 95% air atmosphere at 37 °C. To assess the effects of extracellular acidic microenvironment, the pH of culture medium was adjusted by PIPES and HEPES (Sigma, St. Louis, MO, USA).^48^ To block ASIC1 and ASIC3, amiloride (Sigma) was added to normal or acidic medium with various final concentrations 100 *μ*M for 48 h. To specifically block ASIC1, PcTX1 (Alomone, Jerusalem, Israel) was added to normal or acidic medium with various final concentrations 100 nM for 48 h. To chelate intracellular Ca^2+^, cells were loaded with 100 mM BAPTA-AM (Sigma) for 1 h.

### Patch-clamp recording

The procedure for whole-cell voltage-clamp recording was performed as described previously^42^ with minor modifications. Membrane currents were recorded on PANC-1, BxPC-3 and HPDE with an EPC-10 amplifier (HEKA, Lambrecht, Germany) driven by Pulse/PulseFit software (HEKA). Electrodes (3–5 MΩ) were filled with intracellular solution containing (in mM): 140 KCl, 10 NaCl, 1 MgCl_2_·6H_2_O, 5 EGTA, 2 MgATP, 10 HEPES, pH 7.4 with 1 mM Tris-OH. The whole-cell configuration was established and both PANC-1 and BxPC-3 cells were clamped at −40 mV. A multi-barrel perfusion system was applied to achieve a rapid exchange of extracellular solution (in mM): 5 KCl, 150 NaCl, 1 MgCl_2_·6H_2_O, 2 CaCl_2_, 10 glucose, 10 HEPES, adjusted to pH 7.4 and 6.4 with 1 mM Tris-OH. Whole-cell pH-induced currents were recorded immediately with pH 6.4 extracellular solution application and recorded again after being treated with ASICs inhibitors for 5 min. All experiments were performed at room temperature (20–25 °C).

### Calcium imaging

Intracellular Ca^2+^ concentration [Ca^2+^]i was measured using Fura-2/AM (Gibco, Paisley, Scotland, UK). Cells were seeded on coverslips and incubated with 2 *μ*M Fura-2/AM in Hank’s solution at 37 °C for 30 min. Fluorescence was illuminated at 340 and 380 nm and captured at 1-s intervals by an inverted microscope (Olympus IX-70, Japan), which was equipped with a calcium imaging system (TILL-Photonics, Gräfelfing, German). The emitted light was imaged at 510 nm with a video camera (PTI Image) through an X-70 fluor oil immersion lens (Nikon, Tokyo, Japan) and a 460 nm long-pass barrier filter. The low pH medium was added at the time of 90 s. Paired F340/F380 fluorescence ratio was analyzed with TILL software (version 4.0), which was used as an indicator of [Ca^2+^]i.

### G-LISA RhoA activation assay

RhoA GTPase activity was performed using G-LISA RhoA activation assay kit (Cytoskeleton, Denver, CO, USA) according to the manufacture protocol. In all, 0.5 mg/ml cell lysate protein was added to the wells, and GTP-bound RhoA were bound to wells, which were pre-coated anti-GTP antibody. The bound active RhoA was detected with a RhoA-specific antibody. After incubated with secondary antibody and HRP detection reagents, absorbance was read at 490 nm using a microplate reader.

### Cell transfections

SiRNA duplexes targeting the human ASIC1 or ASIC3, and negative control were synthesized and purified by RiboBio (Ribobio Co., Guangzhou, China). RNA oligonucleotides were transfected using Lipofectamine 2000 (Invitrogen, Carlsbad, CA, USA) at a final concentration of 50 nM according to the manufacturer’s instructions. For stable inhibition of ASIC1 and ASIC3 expression, the sequence of siRNA was structured to lentiviral vector. ASIC1 or ASIC3 overexpression vectors and empty (pcDNA-3.1) vectors were transfected with 1.6 *μ*g for 12-well plates. Cells were cultured at normal medium or acidic medium (pH 6.4) for additional 48 h after transfection until collected for further assay.

### RNA isolation, RT-PCR and qRT-PCR

Total RNA was isolated from either cell lines or tissue samples by Trizol reagent (Invitrogen), then reverse transcribed using reverse transcription kit (Takara, Dalian, China) according to the manufacturer’s instructions. RT-PCR reactions were performed under the following conditions: 94 °C for 3 min; 40 PCR cycles of 94 °C for 30 s, 55 °C for 30 s, 72 °C for 30 s; 72 °C for 10 min. The amplified products were separated by 2% agarose gel electrophoresis. QRT-PCR was performed according to the manufacturer’s protocol (Takara) on Applied Biosystems StepOne-Plus Real-Time PCR System (Thermo Fisher, Wuhan, China) and GAPDH were used to normalize the level of mRNA. Primer sequences used in this study are all shown in Supplementary Table S1.

### Western blot analysis, immunohistochemistry and immunofluorescence

Western blot, Immunohistochemistry and immunofluorescence assay were carried out as previously described.^7^ Antibodies for research were as follows: ASIC1, ASIC3 (Santa Cruz Biotechnology, Shanghai, China); N-cadherin, E-cadherin, vimentin, Snail, ZEB1, RhoA, GAPDH (Cell Signaling, Danvers, MA, USA).

### Cell viability assay

Cell viability was measured by MTT assay after cells were seeded into 96-well plates as a density of 2000 cells per well and incubated at 37 °C for 2 days. The survival rate was calculated as: cell viability=OD570 (experiment group)/OD570 (control group) × 100%. All experiments were repeated three times independently and there were five samples per group.

### Transwell assay and wound-healing assay

For wound-healing assay, the cells were seeded in 12-well plates and were allowed to grow until it reached 90% concentration. The cell monolayer was scratched by micropipette tip and cultured in serum-free medium. Microphotographs were taken at 48 h. For Transwell assay, 24-well Transwell migration chambers (Corning, Shanghai, China) with 8-*μ*m pore polycarbonate membranes was pre-coated with a thin layer of Matrigel Basement Membrane Matrix (BD Biosciences, Shanghai, China). Medium containing 30% fetal bovine serum in the lower chamber was used as chemoattractant. Suspensions of 5 × 10^4^ cells in 100 *μ*l medium were added to the upper chamber and incubated for 48 h. After fixing with 4% paraformaldehyde and staining with 0.1% crystal violet, the number of cells on the membrane of lower chamber was counted under a microscope in nine random fields.

### Pancreatic cancer xenografts

The animal experiments in this study were approved and reviewed by the Animal Research Committee of the Academic Medical Center at Huazhong University of Science and Technology. Care and handling of the animals were in accordance with the guidelines for Institutional and Animal Care and Use Committees. BALB/c female nude mice (4 weeks old) were purchased from Beijing HFK Bio-Technology Co., Ltd (Beijing, China). Mice were randomly divided into three groups with five mice in each group. Transfected BxPC-3 (2 × 10^6^ cells per mouse) cells was suspended in RPMI-1640 medium and injected subcutaneously into the mice. Tumor volumes were measured every 3 days according to the formula V=0.5 × L (length) × W^2^ (width). At 28 days after cell inoculation, mice were killed. Solid tumor tissues were removed and weighed. Tumor metastasis on liver and lung were counted by H&E stain slides.

### Statistical analysis

All data were presented as mean±S.D. Group’s comparisons were analyzed with Student’s *t*-test. The relationships between the expression of ASIC1/ASIC3 and clinical characteristics of pancreatic cancer patients were analyzed using *χ*^2^ tests. *P*<0.05 was considered statistically significant. SPSS 13.0 (IBM, New York, USA) software was used for all statistical analysis.

## References

[bib1] Hidalgo M. Pancreatic cancer. N Engl J Med 2010; 362: 1605–1617. 20427809 10.1056/NEJMra0901557

[bib2] Haeno H, Gonen M, Davis MB, Herman JM, Iacobuzio-Donahue CA, Michor F. Computational modeling of pancreatic cancer reveals kinetics of metastasis suggesting optimum treatment strategies. Cell 2012; 148: 362–375. 22265421 10.1016/j.cell.2011.11.060 PMC3289413

[bib3] Tuveson DA, Neoptolemos JP. Understanding metastasis in pancreatic cancer: a call for new clinical approaches. Cell 2012; 148: 21–23. 22265397 10.1016/j.cell.2011.12.021

[bib4] Polyak K, Weinberg RA. Transitions between epithelial and mesenchymal states: acquisition of malignant and stem cell traits. Nat Rev Cancer 2009; 9: 265–273. 19262571 10.1038/nrc2620

[bib5] Javle MM, Gibbs JF, Iwata KK, Pak Y, Rutledge P, Yu J et al. Epithelial-mesenchymal transition (EMT) and activated extracellular signal-regulated kinase (p-Erk) in surgically resected pancreatic cancer. Ann Surg Oncol 2007; 14: 3527–3533. 17879119 10.1245/s10434-007-9540-3

[bib6] von Burstin J, Eser S, Paul MC, Seidler B, Brandl M, Messer M et al. E-cadherin regulates metastasis of pancreatic cancer *in vivo* and is suppressed by a SNAIL/HDAC1/HDAC2 repressor complex. Gastroenterology 2009; 137: 361–371, 371 e361-365. 19362090 10.1053/j.gastro.2009.04.004

[bib7] Deng S, Zhu S, Wang B, Li X, Liu Y, Qin Q et al. Chronic pancreatitis and pancreatic cancer demonstrate active epithelial-mesenchymal transition profile, regulated by miR-217-SIRT1 pathway. Cancer Lett 2014; 355: 184–191. 25172416 10.1016/j.canlet.2014.08.007

[bib8] Gatenby RA, Gillies RJ. Why do cancers have high aerobic glycolysis? Nat Rev Cancer 2004; 4: 891–899. 15516961 10.1038/nrc1478

[bib9] Schornack PA, Gillies RJ. Contributions of cell metabolism and H+ diffusion to the acidic pH of tumors. Neoplasia (New York, NY) 2003; 5: 135–145. 10.1016/s1476-5586(03)80005-2 PMC1502399 12659686

[bib10] Lindner D, Raghavan D. Intra-tumoural extra-cellular pH: a useful parameter of response to chemotherapy in syngeneic tumour lines. Br J Cancer 2009; 100: 1287–1291. 19367285 10.1038/sj.bjc.6605022 PMC2676543

[bib11] Gillies RJ, Liu Z, Bhujwalla Z. 31P-MRS measurements of extracellular pH of tumors using 3-aminopropylphosphonate. Am J Physiol 1994; 267(1 Pt 1): C195–C203. 8048479 10.1152/ajpcell.1994.267.1.C195

[bib12] van Sluis R, Bhujwalla ZM, Raghunand N, Ballesteros P, Alvarez J, Cerdan S et al. *In vivo* imaging of extracellular pH using 1H MRSI. Magn Reson Med 1999; 41: 743–750. 10332850 10.1002/(sici)1522-2594(199904)41:4<743::aid-mrm13>3.0.co;2-z

[bib13] Peppicelli S, Bianchini F, Torre E, Calorini L. Contribution of acidic melanoma cells undergoing epithelial-to-mesenchymal transition to aggressiveness of non-acidic melanomacells. Clin Exp Metastasis 2014; 31: 423–433. 24469963 10.1007/s10585-014-9637-6

[bib14] Suzuki A, Maeda T, Baba Y, Shimamura K, Kato Y. Acidic extracellular pH promotes epithelial mesenchymal transition in Lewis lung carcinoma model. Cancer Cell Int 2014; 14: 129. 25493076 10.1186/s12935-014-0129-1 PMC4260188

[bib15] Deng S, Li X, Niu Y, Zhu S, Jin Y, Deng S et al. MiR-652 inhibits acidic microenvironment-induced epithelial-mesenchymal transition of pancreatic cancer cells by targeting ZEB1. Oncotarget 2015; 6: 39661–39675. 26498682 10.18632/oncotarget.5350 PMC4741853

[bib16] Waldmann R, Champigny G, Bassilana F, Heurteaux C, Lazdunski M. A proton-gated cation channel involved in acid-sensing. Nature 1997; 386: 173–177. 9062189 10.1038/386173a0

[bib17] Wemmie JA, Price MP, Welsh MJ. Acid-sensing ion channels: advances, questions and therapeutic opportunities. Trends Neurosci 2006; 29: 578–586. 16891000 10.1016/j.tins.2006.06.014

[bib18] Wemmie JA, Taugher RJ, Kreple CJ. Acid-sensing ion channels in pain and disease. Nat Rev Neurosci 2013; 14: 461–471. 23783197 10.1038/nrn3529 PMC4307015

[bib19] Benarroch EE. Acid-sensing cation channels: structure, function, and pathophysiologic implications. Neurology 2014; 82: 628–635. 24443451 10.1212/WNL.0000000000000134

[bib20] Wang YZ, Xu TL. Acidosis, acid-sensing ion channels, and neuronal cell death. Mol Neurobiol 2011; 44: 350–358. 21932071 10.1007/s12035-011-8204-2

[bib21] Krishtal O. The ASICs: signaling molecules? Modulators? Trends Neurosci 2003; 26: 477–483. 12948658 10.1016/S0166-2236(03)00210-8

[bib22] Berdiev BK, Xia J, McLean LA, Markert JM, Gillespie GY, Mapstone TB et al. Acid-sensing ion channels in malignant gliomas. J Biol Chem 2003; 278: 15023–15034. 12584187 10.1074/jbc.M300991200

[bib23] Kapoor N, Bartoszewski R, Qadri YJ, Bebok Z, Bubien JK, Fuller CM et al. Knockdown of ASIC1 and epithelial sodium channel subunits inhibits glioblastoma whole cell current and cell migration. J Biol Chem 2009; 284: 24526–24541. 19561078 10.1074/jbc.M109.037390 PMC2782044

[bib24] Prevarskaya N, Skryma R, Shuba Y. Calcium in tumour metastasis: new roles for known actors. Nat Rev Cancer 2011; 11: 609–618. 21779011 10.1038/nrc3105

[bib25] Gulhati P, Bowen KA, Liu J, Stevens PD, Rychahou PG, Chen M et al. mTORC1 and mTORC2 regulate EMT, motility, and metastasis of colorectal cancer via RhoA and Rac1 signaling pathways. Cancer Res 2011; 71: 3246–3256. 21430067 10.1158/0008-5472.CAN-10-4058 PMC3085654

[bib26] Fernandez-Tenorio M, Porras-Gonzalez C, Castellano A, Del Valle-Rodriguez A, Lopez-Barneo J, Urena J. Metabotropic regulation of RhoA/Rho-associated kinase by L-type Ca2+ channels: new mechanism for depolarization-evoked mammalian arterial contraction. Circ Res 2011; 108: 1348–1357. 21493898 10.1161/CIRCRESAHA.111.240127

[bib27] Webb BA, Chimenti M, Jacobson MP, Barber DL. Dysregulated pH: a perfect storm for cancer progression. Nat Rev Cancer 2011; 11: 671–677. 21833026 10.1038/nrc3110

[bib28] Estrella V, Chen T, Lloyd M, Wojtkowiak J, Cornnell HH, Ibrahim-Hashim A et al. Acidity generated by the tumor microenvironment drives local invasion. Cancer Res 2013; 73: 1524–1535. 23288510 10.1158/0008-5472.CAN-12-2796 PMC3594450

[bib29] Pedersen SF, Stock C. Ion channels and transporters in cancer: pathophysiology, regulation, and clinical potential. Cancer Res 2013; 73: 1658–1661. 23302229 10.1158/0008-5472.CAN-12-4188

[bib30] Becchetti A. Ion channels and transporters in cancer. 1. Ion channels and cell proliferation in cancer. Am J Physiol Cell Physiol 2011; 301: C255–C265. 21430288 10.1152/ajpcell.00047.2011

[bib31] Schwab A, Fabian A, Hanley PJ, Stock C. Role of ion channels and transporters in cell migration. Physiol Rev 2012; 92: 1865–1913. 23073633 10.1152/physrev.00018.2011

[bib32] Stock C, Schwab A. Ion channels and transporters in metastasis. Biochim Biophys Acta 2015; 1848(10 Pt B): 2638–2646. 25445667 10.1016/j.bbamem.2014.11.012

[bib33] Gupta SC, Singh R, Asters M, Liu J, Zhang X, Pabbidi MR et al. Regulation of breast tumorigenesis through acid sensors. Oncogene 2016; 35: 4102–4111. 26686084 10.1038/onc.2015.477 PMC6450404

[bib34] Jin C, Ye QH, Yuan FL, Gu YL, Li JP, Shi YH et al. Involvement of acid-sensing ion channel 1alpha in hepatic carcinoma cell migration and invasion. Tumour Biol 2015; 36: 4309–4317. 25613068 10.1007/s13277-015-3070-6

[bib35] Li Y, Xu G, Huang K, Wang J, Zhang J, Liu J et al. Alteration of ASIC1 expression in clear cell renal cell carcinoma. OncoTargets Ther 2015; 8: 2121–2127. 10.2147/OTT.S86927 PMC4542551 26316781

[bib36] Cullen PJ, Lockyer PJ. Integration of calcium and Ras signalling. Nat Rev Mol Cell Biol 2002; 3: 339–348. 11988768 10.1038/nrm808

[bib37] Monteith GR, Davis FM, Roberts-Thomson SJ. Calcium channels and pumps in cancer: changes and consequences. J Biol Chem 2012; 287: 31666–31673. 22822055 10.1074/jbc.R112.343061 PMC3442501

[bib38] Monet M, Lehen'kyi V, Gackiere F, Firlej V, Vandenberghe M, Roudbaraki M et al. Role of cationic channel TRPV2 in promoting prostate cancer migration and progression to androgen resistance. Cancer Res 2010; 70: 1225–1235. 20103638 10.1158/0008-5472.CAN-09-2205

[bib39] Davis FM, Azimi I, Faville RA, Peters AA, Jalink K, Putney JW et al. Induction of epithelial–mesenchymal transition (EMT) in breast cancer cells is calcium signal dependent. Oncogene 2013; 33: 2307–2316. 23686305 10.1038/onc.2013.187 PMC3917976

[bib40] Gong W, Kolker SJ, Usachev Y, Walder RY, Boyle DL, Firestein GS et al. Acid-sensing ion channel 3 decreases phosphorylation of extracellular signal-regulated kinases and induces synoviocyte cell death by increasing intracellular calcium. Arthritis Res Ther 2014; 16: R121. 24923411 10.1186/ar4577 PMC4095605

[bib41] Yermolaieva O, Leonard AS, Schnizler MK, Abboud FM, Welsh MJ. Extracellular acidosis increases neuronal cell calcium by activating acid-sensing ion channel 1a. Proc Natl Acad Sci USA 2004; 101: 6752–6757. 15082829 10.1073/pnas.0308636100 PMC404117

[bib42] Yu X-W, Hu Z-L, Ni M, Fang P, Zhang P-W, Shu Q et al. Acid-sensing ion channels promote the inflammation and migration of cultured rat microglia. Glia 2015; 63: 483–496. 25377529 10.1002/glia.22766

[bib43] Jaffe AB, Hall A. Rho GTPases: biochemistry and biology. Ann Rev Cell Dev Biol 2005; 21: 247–269. 16212495 10.1146/annurev.cellbio.21.020604.150721

[bib44] Rao JN, Li L, Golovina VA, Platoshyn O, Strauch ED, Yuan JX et al. Ca2+-RhoA signaling pathway required for polyamine-dependent intestinal epithelial cell migration. Am J Physiol Cell Physiol 2001; 280: C993–1007. 11245616 10.1152/ajpcell.2001.280.4.C993

[bib45] Li H, Wang Z, Zhang W, Qian K, Xu W, Zhang S. Fbxw7 regulates tumor apoptosis, growth arrest and the epithelial-to-mesenchymal transition in part through the RhoA signaling pathway in gastric cancer. Cancer Lett 2016; 370: 39–55. 26458995 10.1016/j.canlet.2015.10.006

[bib46] Lin S, Gregory RI. MicroRNA biogenesis pathways in cancer. Nat Rev Cancer 2015; 15: 321–333. 25998712 10.1038/nrc3932 PMC4859809

[bib47] Vega FM, Ridley AJ. Rho GTPases in cancer cell biology. FEBS Lett 2008; 582: 2093–2101. 18460342 10.1016/j.febslet.2008.04.039

[bib48] Rofstad EK, Mathiesen B, Kindem K, Galappathi K. Acidic extracellular pH promotes experimental metastasis of human melanoma cells in athymic nude mice. Cancer Res 2006; 66: 6699–6707. 16818644 10.1158/0008-5472.CAN-06-0983

