# Supplementary material for: ASIC1 and ASIC3 contribute to acidity-induced EMT of pancreatic cancer through activating Ca2+/RhoA pathway
Source: Cell Death Dis. 2017 May 18;8(5):e2806–. doi: 10.1038/cddis.2017.189 (PMC5520710; doi:10.1038/cddis.2017.189)
Supplement: Supplementary Figure Legends [file cddis2017189x1.docx]

**Supplementary figure legends**

**Supplementary Figure S1. Acidic solution did not induce inward currents in normal pancreatic ductal cells**. (A) Whole-cell current of normal pancreatic ductal cells (HPDE) incubated with acidic (pH6.4) extracellular solution was recorded by whole-cell voltage-clamp recording.

**Supplementary Figure S2. SiRNA of ASIC1 and ASIC3 significant suppress expression of ASIC1 and ASIC3.** (A) qRT-PCR and Western blot showed mRNA and protein expression of ASIC1 after PANC-1 and BxPC-3 were transfected with ASIC1 siRNA(si-1, si-2, si-3). (B) qRT-PCR and Western blot showed mRNA and protein expression of ASIC3 after PANC-1 and BxPC-3 were transfected with ASIC3 siRNA(si-1, si-2, si-3). (C) qRT-PCR and Western blot showed mRNA and protein expression of ASIC1 and ASIC3 after PANC-1 and BxPC-3 were transfected with ASIC1 siRNA #2(siASIC1) and ASIC3 siRNA #2(siASIC3) separately or simultaneously. Values were normalized against negative control. Experiments were performed three times in triplicate and are presented as means ± SD (*, P< 0.05; **, P<0.01).

**Supplementary Figure S3. PcTX1 suppressed the acidity-induced invasion and migration.** PANC-1 and BxPC-3 were treated with or without PcTX1, and cultured in pH7.4 or pH6.4 medium as indicated for 48 hours. (A) The invasive ability was evaluated by Transwell assay. The histogram showed the percentage of invasion cells per field compared to control cultured in pH6.4 medium. (B) The migration ability was measured by Wound healing assay. Experiments were performed three times in triplicate and are presented as means ± SD (*, P< 0.05; **, P<0.01).

**Supplementary Figure S4. Inhibition of ASIC1 and ASIC3 impairs** **acidity-induced mesenchymal profile.** (A) Cellular morphology of PANC-1 and BxPC-3 which were transfected with negative control (NC) or siRNA of ASIC1/ASIC3 (siASIC1, siASIC3) and cultured in pH7.4 or pH6.4 medium as indicated for 48 hours. (B) Cellular morphology of PANC-1 and BxPC-3 which were treated with amiloride or DMSO and cultured in pH7.4 or pH6.4 medium as indicated for 48 hours.

**Supplementary Figure S5. PcTX1 suppressed the acidity-induced EMT.** PANC-1 and BxPC-3 were treated with or without PcTX1, and cultured in pH7.4 or pH6.4 medium as indicated for 48 hours. (A) The mRNA of N-cadherin, E-cadherin, Vimentin, ZEB1, Snail was measured by qRT-PCR. (B) The protein of N-cadherin, E-cadherin, Vimentin, ZEB1, Snail was measured by Western blot. Values were normalized against control in pH 6.4 medium. Experiments were performed three times in triplicate and are presented as means ± SD (*, P< 0.05; **, P<0.01; ***, P<0.001).

**Supplementary Figure S6. Overexpression of ASIC1 or ASIC3 promoted EMT of AsPC-1 in acidic condition.** AsPC-1 cells were transfected with ASIC1 overexpression vector (ASIC1-OE), ASIC3 overexpression vector （ASIC3-OE）and empty vector （Vector）as negative control. The transfected cells were cultured in acidic (pH=6.4) condition for 48 hours. (A) Western blot showed protein of ASIC1 and ASIC3. (B) The invasive ability was evaluated by Transwell assay. The histogram showed the percentage of invasion cells per field compared to negative control. (C) The migration ability was measured by Wound healing assay. Values were normalized against negative control. (D) The mRNA of N-cadherin, E-cadherin, Vimentin, ZEB1, Snail was measured by qRT-PCR. (E) The protein of N-cadherin, E-cadherin, Vimentin, ZEB1, Snail was measured by Western blot. Values were normalized against negative control. Experiments were performed three times in triplicate and are presented as means ± SD (*, P< 0.05; **, P<0.01; ***, P<0.001).

**Supplementary Figure S7. The acidity-induced EMT is suppressed by BAPTA-AM.** PANC-1 and BxPC-3 were treated with BAPTA-AM or DMSO for 1 hour and cultured in pH7.4 or pH6.4 medium as indicated for 48 hours. (A) The invasive ability was evaluated by Transwell assay. The histogram showed the percentage of invasion cells per field compared to negative control cultured in pH6.4 medium. (B) The migration ability was measured by Wound healing assay. (C) The representative picture of cellular morphology. (D) The mRNA of N-cadherin, E-cadherin, Vimentin, ZEB1, Snail was measured by qRT-PCR. (E) The protein of N-cadherin, E-cadherin, Vimentin, ZEB1, Snail was measured by Western blot. Values were normalized against DMSO group in pH 6.4 medium. Experiments were performed three times in triplicate and are presented as means ± SD (*, P< 0.05; **, P<0.01; ***, P<0.001).

**Supplementary Figure S8. SiRNA of RhoA significantly suppresses expression of RhoA.** (A) qRT-PCR and Western blot showed mRNA and protein of RhoA after PANC-1 and BxPC-3 were transfected with siRNA of RhoA(si-1, si-2, si-3). Values were normalized against negative control. Experiments were performed three times in triplicate and are presented as means ± SD (*, P< 0.05; **, P<0.01).
