# Supplementary material for: ASIC1 and ASIC3 contribute to acidity-induced EMT of pancreatic cancer through activating Ca2+/RhoA pathway
Source: Cell Death Dis. 2017 May 18;8(5):e2806–. doi: 10.1038/cddis.2017.189 (PMC5520710; doi:10.1038/cddis.2017.189)
Supplement: Supplementary Table S1 [file cddis2017189x2.docx]

**Supplementary Table S1.** The sequence of PCR primers.

| **Primer** | **Sequence** |
| --- | --- |
| E-cadherin | forward: 5’-GACAACAAGCCCGAATT-3’ |
|  | reverse: 5’-GGAAACTCTCTCGGTCCA-3’ |
| N-cadherin | forward: 5’-CTCCTATGAGTGGAACAGGAACG-3’ |
|  | reverse: 5’-TTGGATCAATGCATAATCAAGTGCTGTA-3’ |
| Snail | forward: 5’-GAGGCGGTGGCAGACT-3’ |
|  | reverse: 5’-GACACATCGGTCAGACCAG-3’ |
| ZEB1 | forward: 5’-AAGAATTCACAGTGGAGAGAAGCCA-3’ |
|  | reverse: 5’-CGTTT CTTGCAGTTTGGGCATT-3’ |
| Vimentin | forward: 5’-GGACCAGCTAACCAACGACA-3’ |
|  | reverse: 5’-AAGGTCAAGACGTGCCAGAG-3’ |
| ASIC1 | forward: 5’-CAGTGGCGACAAAGGAAG-3’ |
|  | reverse: 5’-AATGGGATAGGAGGAGCAG |
| ASIC3 | forward: 5’-TTCTGGAACCGACAGCACTC-3’ |
|  | reverse: 5’-TGGGGCCCAGGCTGA-3’ |
| GAPDH | forward: 5’- TGAACGGGAAGCTCACTGG-3’ |
|  | reverse: 5’- TCCACCACCCTGTTGCTGTA-3’ |
