# Supplementary material for: ASIC1 and ASIC3 contribute to acidity-induced EMT of pancreatic cancer through activating Ca2+/RhoA pathway
Source: Cell Death Dis. 2017 May 18;8(5):e2806–. doi: 10.1038/cddis.2017.189 (PMC5520710; doi:10.1038/cddis.2017.189)
Supplement: Supplementary Table S2 [file cddis2017189x3.docx]

|  | **ASIC1 expression** | |  | **P** |
| --- | --- | --- | --- | --- |
|  | High | Low | Total |  |
| **Genda** |  |  |  |  |
| Male | 14 | 13 | 27 | 1 |
| Femal | 6 | 7 | 13 |  |
| **Age** |  |  |  |  |
| <60 | 9 | 5 | 14 | 0.32 |
| ≥60 | 11 | 15 | 26 |  |
| **Tumor size** |  |  |  |  |
| < 2cm | 7 | 9 | 16 | 0.748 |
| ≥ 2cm | 13 | 11 | 24 |  |
| **Tumor differentiation** |  |  |  |  |
| Well | 7 | 15 | 22 | 0.025* |
| Poor/Moderate | 13 | 5 | 18 |  |
| **TNM stage** |  |  |  |  |
| I~II | 4 | 12 | 16 | 0.023* |
| III~IV | 16 | 8 | 24 |  |
| **Lymphatic metastasis** |  |  |  |  |
| Positive | 14 | 6 | 20 | 0.026* |
| Negative | 6 | 14 | 20 |  |
| **Distant metastasis** |  |  |  |  |
| Positive | 15 | 6 | 21 | 0.010* |
| Negative | 5 | 14 | 19 |  |

Supplementary Table S2. Correlation between ASIC1 expression and clinical characteristics of patient with pancreatic cancer.
