# Supplementary material for: ASIC1 and ASIC3 contribute to acidity-induced EMT of pancreatic cancer through activating Ca2+/RhoA pathway
Source: Cell Death Dis. 2017 May 18;8(5):e2806–. doi: 10.1038/cddis.2017.189 (PMC5520710; doi:10.1038/cddis.2017.189)
Supplement: Supplementary Table S3 [file cddis2017189x4.docx]

|  | **ASIC3 expression** | |  | **P** |
| --- | --- | --- | --- | --- |
|  | High | Low | Total |  |
| **Genda** |  |  |  |  |
| Male | 12 | 15 | 27 | 0.5 |
| Femal | 8 | 5 | 13 |  |
| **Age** |  |  |  |  |
| <60 | 10 | 8 | 18 | 0.751 |
| ≥60 | 10 | 12 | 22 |  |
| **Tumor size** |  |  |  |  |
| < 2cm | 9 | 8 | 16 | 1 |
| ≥ 2cm | 11 | 12 | 24 |  |
| **Tumor differentiation** |  |  |  |  |
| Well | 4 | 12 | 16 | 0.022* |
| Poor/Moderate | 16 | 8 | 24 |  |
| **TNM stage** |  |  |  |  |
| I~II | 5 | 14 | 19 | 0.010* |
| III~IV | 15 | 6 | 21 |  |
| **Lymphatic metastasis** |  |  |  |  |
| Positive | 13 | 3 | 16 | 0.003* |
| Negative | 7 | 17 | 24 |  |
| **Distant metastasis** |  |  |  |  |
| Positive | 14 | 5 | 19 | 0.010* |
| Negative | 6 | 15 | 21 |  |

Supplementary Table S3. Correlation between ASIC3 expression and clinical characteristics of patient with pancreatic cancer.
