# Supplementary figures and images for: ASIC1 and ASIC3 contribute to acidity-induced EMT of pancreatic cancer through activating Ca2+/RhoA pathway
Source: Cell Death Dis. 2017 May 18;8(5):e2806–. doi: 10.1038/cddis.2017.189 (PMC5520710; doi:10.1038/cddis.2017.189)

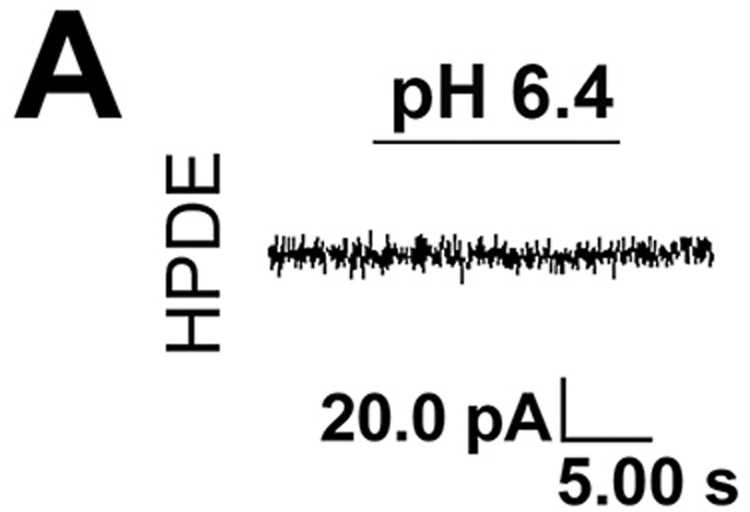

Supplement: Supplementary Figure S1 [file cddis2017189x5.tif]

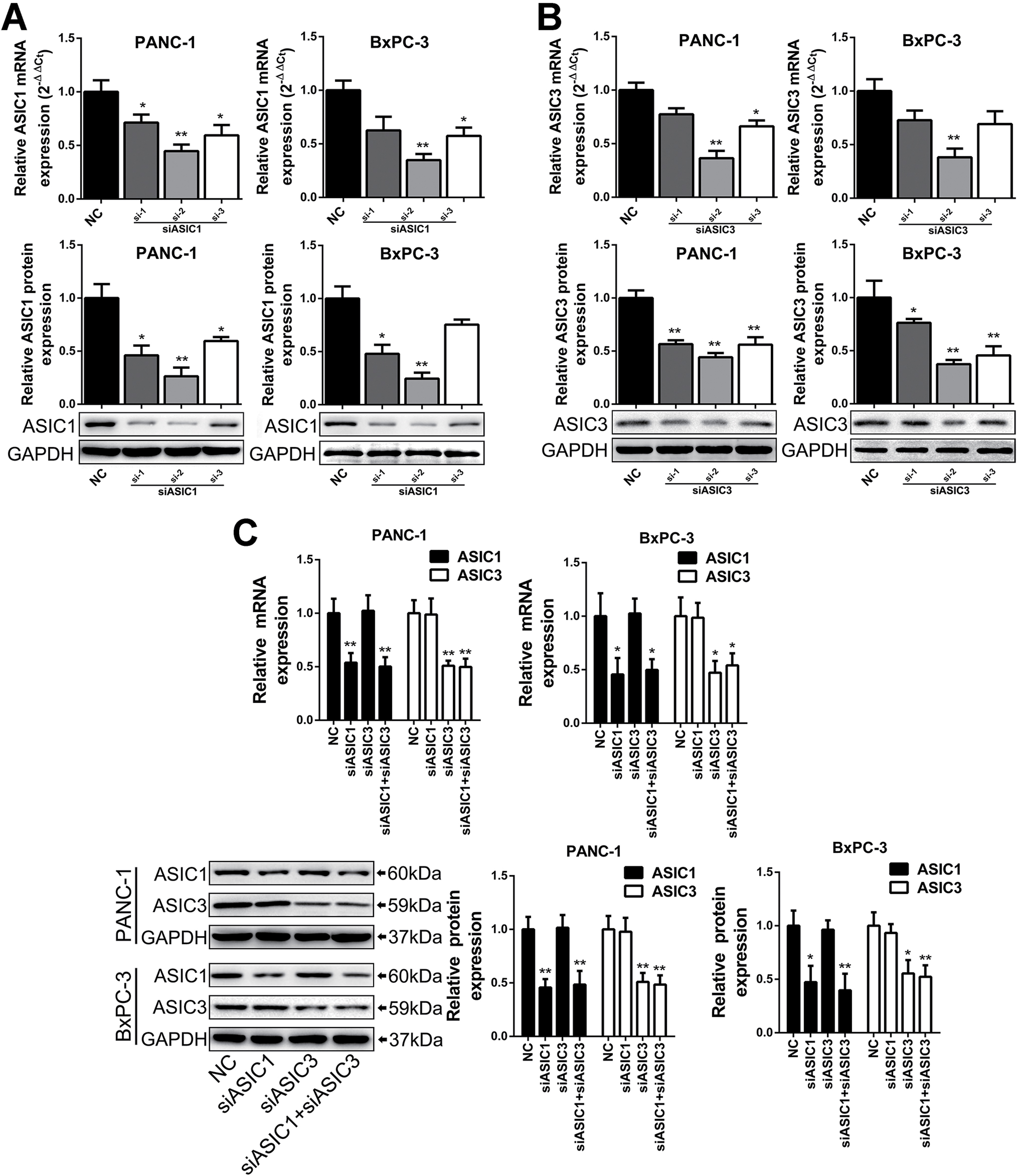

Supplement: Supplementary Figure S2 [file cddis2017189x6.tif]

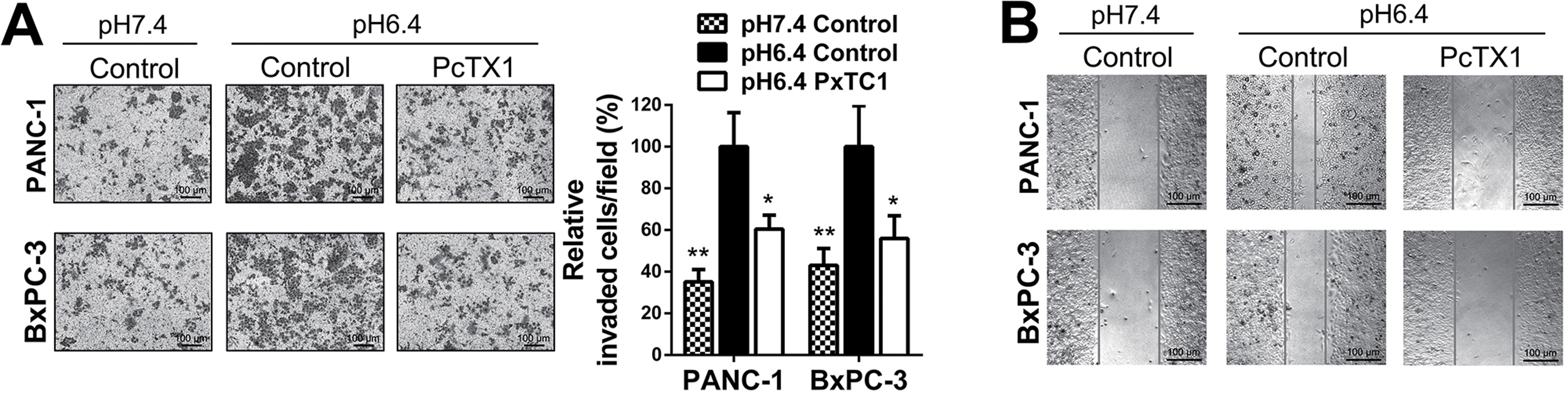

Supplement: Supplementary Figure S3 [file cddis2017189x7.tif]

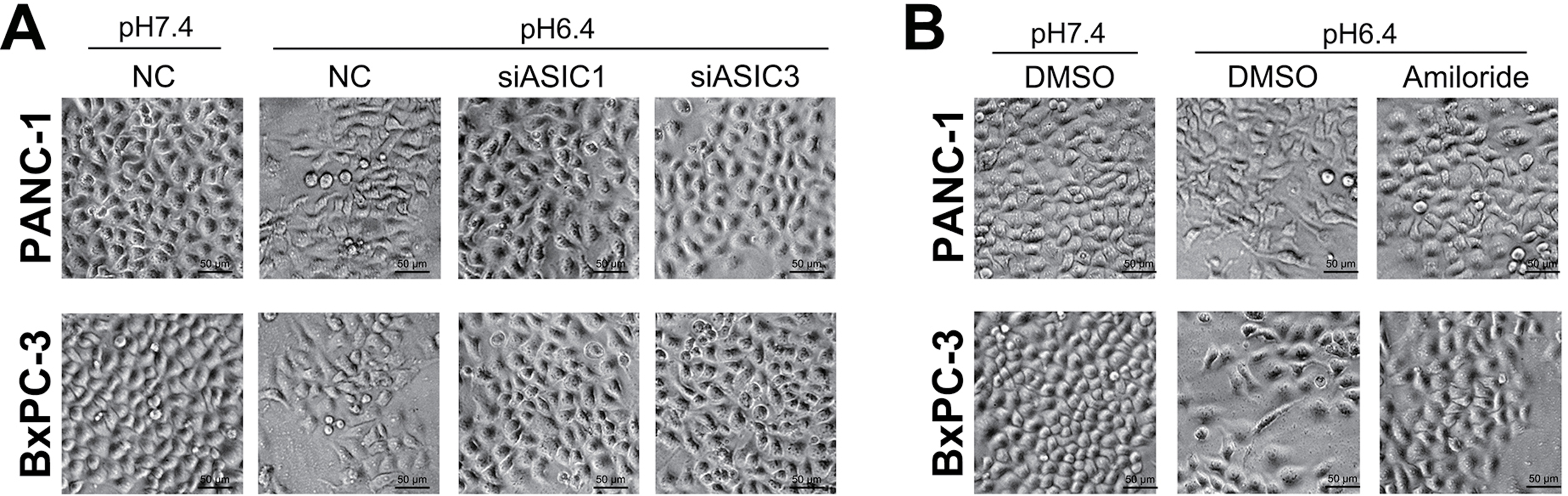

Supplement: Supplementary Figure S4 [file cddis2017189x8.tif]

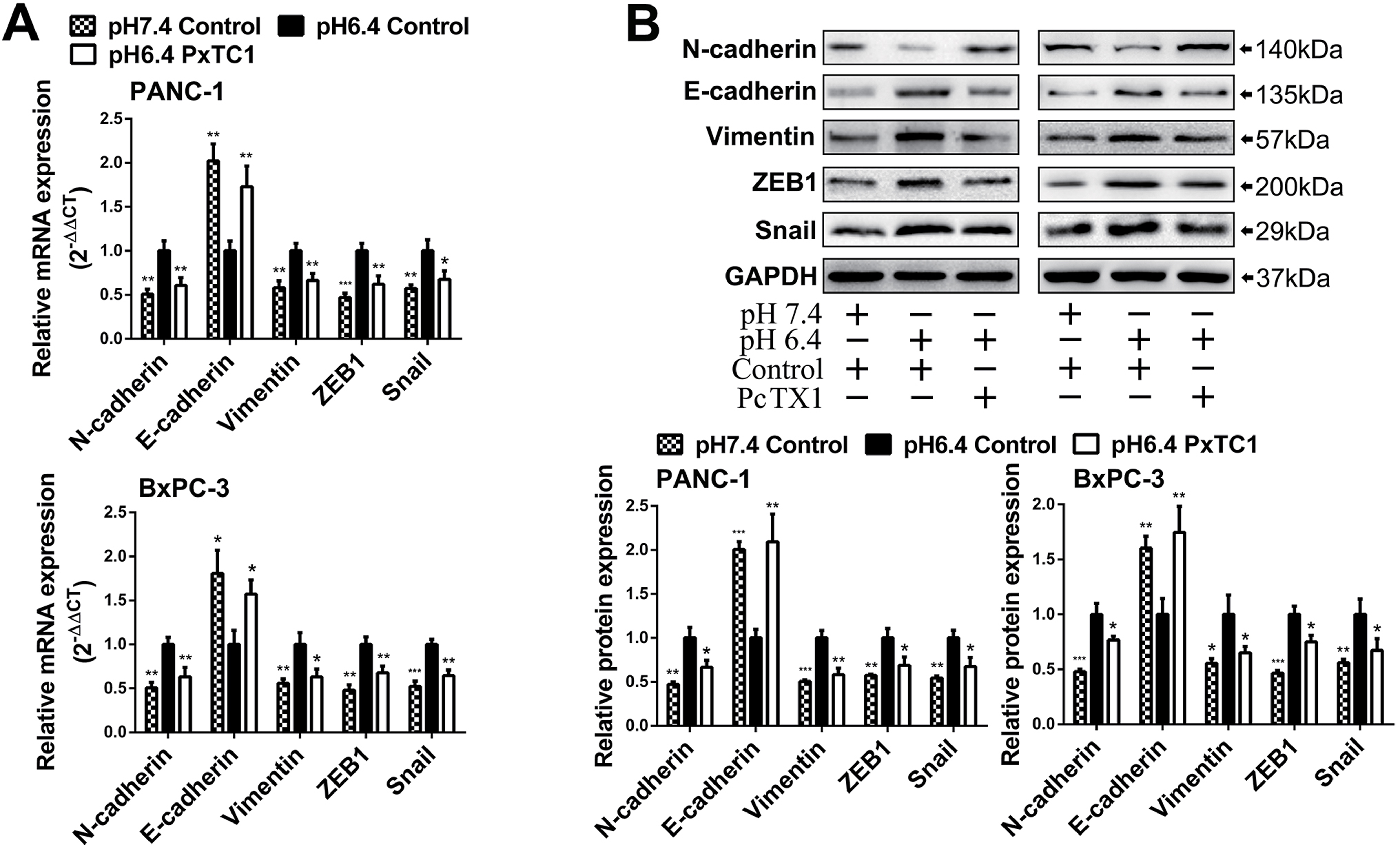

Supplement: Supplementary Figure S5 [file cddis2017189x9.tif]

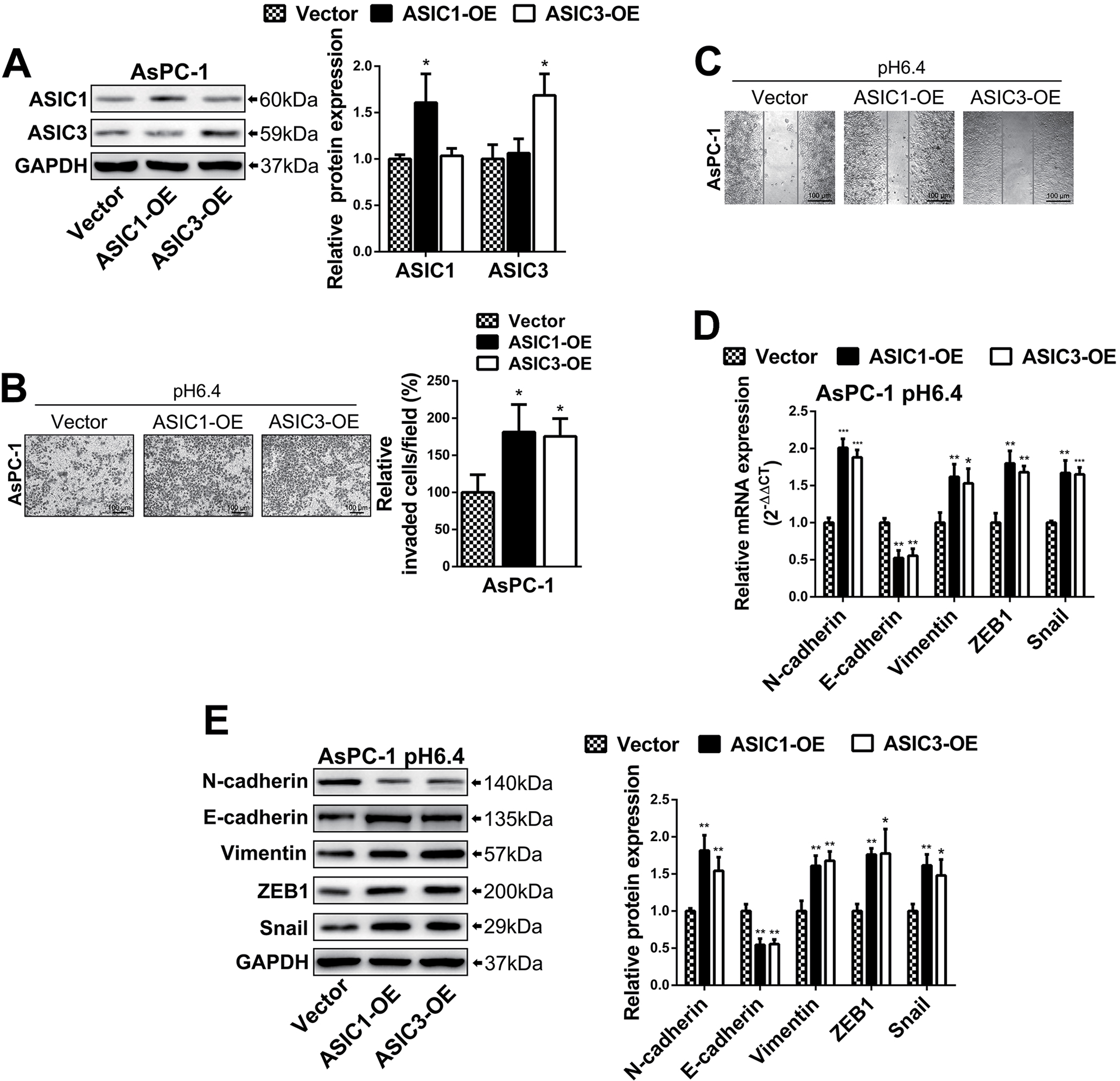

Supplement: Supplementary Figure S6 [file cddis2017189x10.tif]

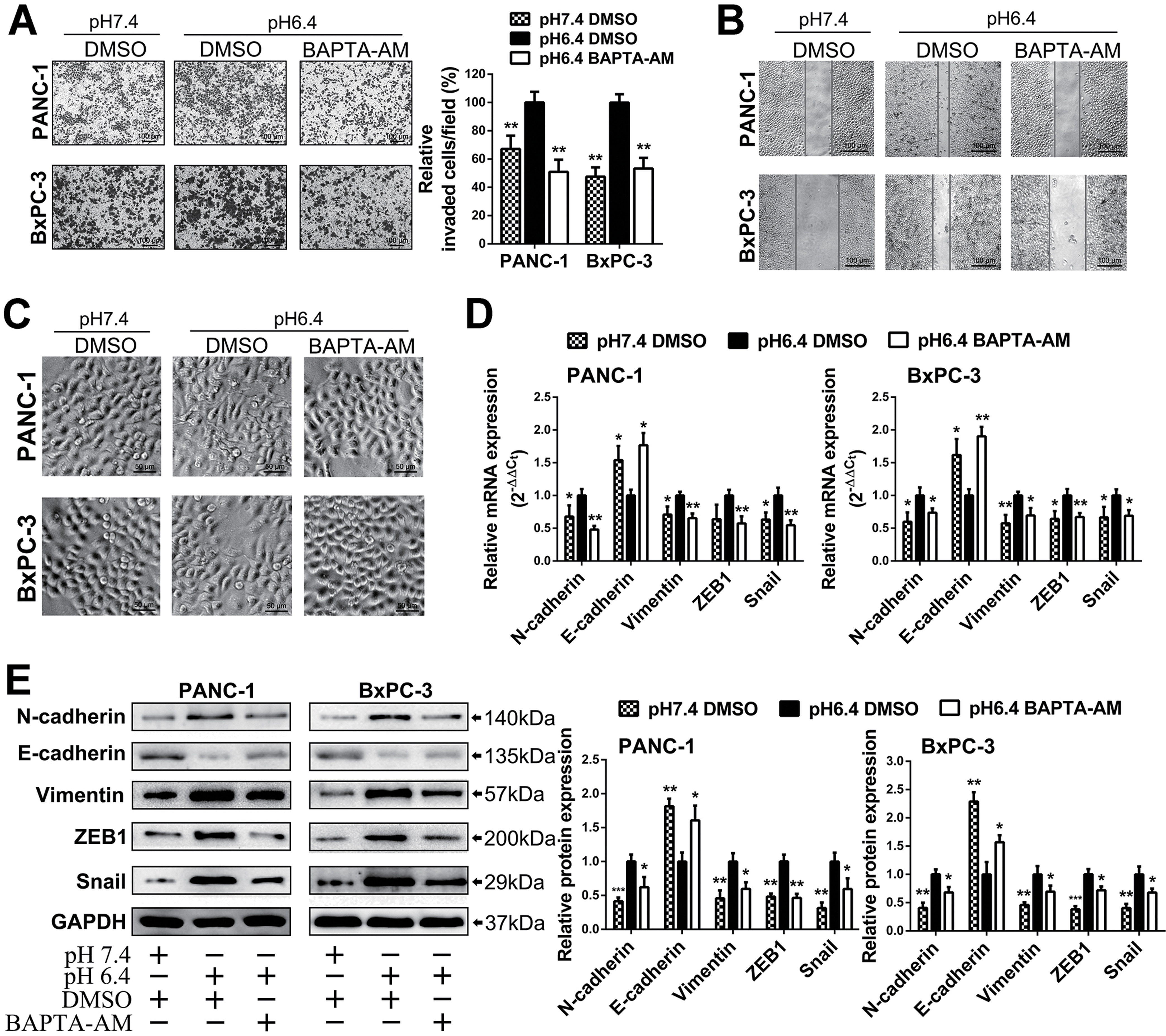

Supplement: Supplementary Figure S7 [file cddis2017189x11.tif]

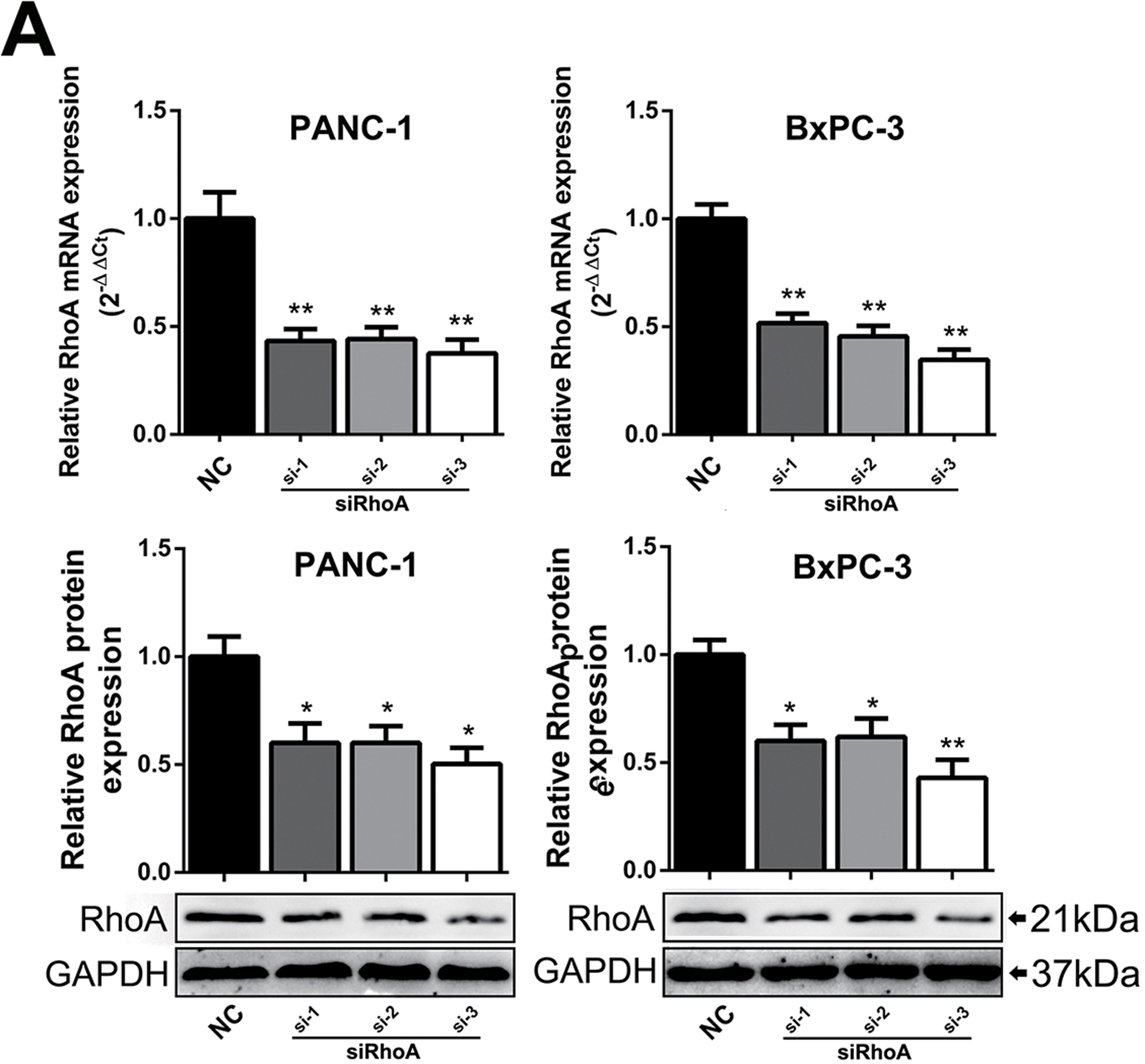

Supplement: Supplementary Figure S8 [file cddis2017189x12.tif]
